# Round the Hospitals

**Published:** 1921-03-12

**Authors:** 


### ROUND THE HOSPITALS.

One of the great pioneers has passed to her rest in the person of Miss Lucy Robinson, who died on February 25 at the age of sixty-seven, and was buried in Brompton Cemetery. For a great part of her life she bent her energies to rescue the calling of the masseuse from the poor esteem in which it was held in England. She was one of the twelve founder-members who set the Incorporated Society of Trained Masseuses on its feet in 1894, and she had the happiness before her death of seeing it expand, as a consequence of her endeavours, into the Chartered Society of Massage and Medical Gymnastics. Her qualifications as a masseuse were the highest obtainable in this country. It was through her persistent energy that permission was gained for masseurs and masseuses to take the anatomy course at the London School of Medicine, and she herself was the first to do so. She was a member of the Advisory Massage Committee to the War Office during the war, and was one of the Chartered Society's representatives on the similar Committee in connection with the Ministry of Pensions. In her single-hearted pursuit of the one object, the bettering of her profession, she is worthy of high honour and ever-grateful remembrance. It is singular that her death almost coincided with that of Mrs. Palmer, another founder-member of the I.S.T.M., whose "Lessons on Massage" are widely known. Miss Robinson, in addition to her work for masseuses, helped to found the Association for Promoting the Training and Employment of Midwives. She was a member of the Council of the Midwives' Institute, and took an active part in its work.

It will be seen from the report of the proceedings on page 548, that the General Nursing Council for Ireland has formulated rules for the registration of "existing nurses." The minimum standard adopted, after taking legal advice, is one year's hospital training, and it was agreed unanimously that nurses who have been employed in Poor-law institutions as "qualified nurses" within the meaning of the Local Government Board Act (1901) are to be eligible for admission to the Register. No one will grudge any benefit conferred by registration on this particularly ill-paid and overworked group of women. But their admission as registered nurses may prove a test of Irish devotion to the principle of registration. It has been decided by the Irish Nursing Council that nurses must be registered in the country in which they were trained. We are glad to note the recommendation that there shall be but one General Nursing Council for the whole of Ireland.

A very unhappy state of affairs has prevailed for some time past at the Portsmouth Infirmary, owing, so the Guardians contend, to continued friction

**ROUND THE HOSPITALS**—(*continued*).

between the Medical Superintendent and the Matron. Having requested the Ministry of Health to hold a public inquiry into the circumstances, the Guardians have received in reply a request for clear and definite information. The report which the Guardians had already forwarded was read in the presence of the press. It contained a lengthy account of alleged disagreements between the two officials, and concluded with a declaration that "the majority of the Guardians have unquestionably lost confidence in the Medical Superintendent and the Matron." The Ministry of Health is to be asked to receive a deputation from the Board.

---

The Knaresborough Guardians are greatly troubled by the restlessness of the modern young woman and her habit of coming and going instead of remaining to take a settled course. The usual remedy of Guardians is to make what they term "a clean sweep" when confronted with discontent in their nurses. But when this policy is applied to a staff with serious grievances it is apt to lead to more "unpleasantness" than it remedies. The simple method of investigating complaints as a prelude to redressing grievances has never been known to fail.

---

The pleasing manner in which gifts provoke gifts has often been remarked. The latest instance is the handsome Carnegie gift of £500 towards providing books for the beautiful panelled library at the Cowdray Club. The library is one of the choicest rooms in the mansion, and when the shelves are filled with a well-chosen collection, consisting not entirely of professional works, it will be a centre of interest to all who visit the Club. Incidentally we congratulate members on the generous offer of free aural advice which is now within their reach. Mr. Archer Ryland, F.R.C.S., has kindly offered to give advice to any College members needing the services of an ear, nose, and throat specialist, who make application through the Secretary to the College.

---

A special women's department is being formed at the Dudley Road Hospital under the Birmingham Guardians. This is a novelty in that it will approximate to the aims of voluntary hospitals in recognising the need for prevention as well as for alleviation. It will be an important centre for research in diseases peculiar to women, with a consultant's laboratory attached. Twelve wards, including infectious and maternity blocks, have been re-opened at the hospital, and occupied in each case within a few days of the opening, with a total of 440 patients in residence. One of its most important functions is as a training-centre for nurses and midwives. The births in Birmingham total about 20,000 a year. In 1919 there were 744 still-born infants in the city, and 1,630 infants who died in their first year. The number of women who died of puerperal fever was thirty, and as many again died of accidents in child-birth.

A correspondent sends us an appreciation of nurses from the *Daily Mail*, headed "Woman at Her Best," by Alfred Edye, on the ground that such recognition, "all too rare, is worth a place in the nurses' own paper." The writer is lost in admiration at the nurse's efficiency. "You will see a mere slip of a girl, pretty and delicately nurtured, take charge of a difficult case. In a few hours she has a strong man entirely helpless in her hands." "She will stand no nonsense from him—there is the iron hand beneath the velvet glove," and so on, till we reach the culminating reflection: "What should we do without women when we are lying on our beds of pain?" One word of business appreciation, we confess, would have satisfied us better than these pretty sentiments. Unless men will take a little trouble to see that nurses get a living wage, the time will come when there will be beds of pain and no trained nurses at hand to smooth the pillow.

---

The Guild of Health held one of its periodical meetings on Monday, March 7, at the Church House, Westminster. The subject of the addresses was Christian Psychology and Spiritual Healing, and the speakers (the Bishops of Kensington and St. Albans, Miss Eleanor Reed, and the Rev. Harold Anson) addressed themselves to the need for the application of Christ's teaching through the study of psychology to everyday life. The need of the day is to think rightly, to live simply, to exorcise fear, and to cultivate faith as opposed to credulity. Here is a field for the trained nurse, who in attendance on her patient has unusual opportunity to direct their thoughts to the right channels. The Guild of Health, in promoting the exercise of healing by spiritual means, works in complete loyalty to scientific principles and methods. It recognises the power of medicine and the need for alliance between it and spiritual means in the cultivation of both individual and corporate health. It has been established for seventeen years, and the head office is at 3 Bedford Square, W.C. 1., where we advise all who are interested to write for particulars. The Bishop of Kensington is the President and the Rev. Harold Anson the Chairman. The Bishop of St. Albans, at Monday's meeting, made some trenchant comments, which seemed to be echoed by his audience, on the teaching of religion to children, the divorce between religion and life and the bogey of fear on which he declared our social system is based.

---

The Nurses' Missionary League is having a Day of Thought and Prayer for its members on Friday, March 18. It will be held at University Hall, Gordon Square, W.C., and conducted by the Rev. E. A. Miller, formerly chaplain of Bradford Royal Infirmary. The morning session lasts from 10.30 to 12.15, and Miss Sparshott has kindly consented to come from Manchester to give an address on "Fear." The afternoon session is from 3 to 4.30, with an address by Miss A. E. Macdonald on "The Spirit of Service: The Sphere and Influence of the Private Nurse," and the evening session is from

**ROUND THE HOSPITALS**—(*continued*).

7.30 to 8.30, with an address by the Rev. E. A. Miller on "Discipleship." At each session there will be a devotional address, followed by intercession for nurses in various branches of work. Tea will be served at 4.30.

In New Zealand the practice of massage has been placed on a sound footing by the Act providing for the registration of masseurs, which came into force on January 1, 1921. Under this Act, which is administered by a Registration Board consisting of the Registrar (the Inspector-General of Hospitals), a person engaged in the practice of massage in New Zealand, and a registered medical practitioner, who are appointed for three years, no unregistered person may, after January 1, 1923, be employed or continue to be employed as a masseur in any public hospital or other public institution. The qualifications for registration are (*a*) the practice of massage for not less than three years during the five years immediately preceding the commencement of the Act; or (*b*) satisfactory training and the possession of a certificate granted after examination and recognised by the Registrar; or (*c*) the passing of an examination under the Act after a course of not less than six months' training in theoretical massage at an approved school for massage, and not less than six months' training in practical massage at an approved public hospital or other institution in the case of persons registered under the Nurses' Registration Act, 1908, or not less than twelve months' such training in any other case. No persons will be allowed to register under (*a*) after the expiration of two years from the commencement of the Act. It is an offence which is punishable by a fine not exceeding £20 for an unregistered person to claim to be registered under the Act. The term "masseur" in the Act, of course, includes a masseuse. We congratulate the profession of massage in New Zealand that this recognition has been granted to them under an Act which makes registration compulsory and thus affords real protection to the public.

Clayton Hospital, Wakefield, has suffered a heavy loss in the death of its matron, Miss T. M. Pressland, who for the past nineteen years has held office there and played a considerable part in the many changes and improvements which this hospital has seen. Miss Pressland took a great interest in the development of her profession, and was secretary of the Yorkshire Group of the Association of Hospital Matrons and Local Representative for the College of Nursing Yorkshire Centre, by whose members she will be much missed. She was trained at King Edward VII. Hospital, Cardiff, and gained invaluable experience for her duties at Wakefield, first as assistant matron at the Royal Victoria Infirmary, Newcastle, and later as matron of the Durham County Hospital. Miss Pressland died on March 4, and her funeral took place on the 8th instant at Wakefield. A special service was held in the Cathedral.

